# The complete mitochondrial genome of *Echinolaelaps fukienensis* provide insights into phylogeny and rearrangement in the superfamily Dermanyssoidea

**DOI:** 10.1371/journal.pone.0288991

**Published:** 2023-12-15

**Authors:** Gangxian He, Wei Li, Bili Yuan, Wenge Dong

**Affiliations:** 1 Institute of Pathogens and Vectors, Yunnan Provincial Key Laboratory for Zoonosis Control and Prevention, Dali University, Dali, Yunnan, China; 2 Asset and Laboratory Management Office, Dali University, Dali, Yunnan, China; Central University of Punjab, INDIA

## Abstract

**Background:**

*Echinolaelaps fukienensis* is the dominant mite species parasitic on the body surface of the genus *Niviventer*. The mitochondrial genome (mitogenome) has its own independent genetic material and genetic system, and is now widely used in population genetics, genealogical biogeography, phylogeny and molecular evolution studies. Species diversity of the superfamily Dermanyssoidea is very rich, but its mitogenomes AT content is high, and it is difficult to amplify the complete mitogenome by routine PCR. To date, we have only obtained the mitogenomes of 6 species, scarcity on sequence data has greatly impeded the studies in the superfamily Dermanyssoidea.

**Methods:**

*Echinolaelaps fukienensis* were collected in 2019 from the body surface of *Niviventer confucianus* (Rodentia, Muridae) in Yunnan Province. The *E*. *fukienensis* mitogenome was determined and analyzed for the first time using the Illumina Novoseq 6000 platform. Phylogenetic analyses of the superfamily Dermanyssoidea were conducted based on the entire mitogenome sequences.

**Results:**

The *E*. *fukienensis* mitogenome was 14,402 bp, which is known the smallest genome of the superfamily Dermanyssoidea, encoding a total of 37 genes, including 13 PCGs, 22 tRNAs, 2 rRNAs and 1 control region. Most protein-coding genes use ATN as the start codon and TAN as the stop codon. AT and GC skew of *atp8* genes in *E*. *fukienensis* were both 0. The average length of 22 tRNA genes of *E*. *fukienensis* was 64 bp, and secondary structures of tRNAs showed base mismatches and missing D-arms in many places. Compared with gene arrangement pattern of the hypothetical ancestor of arthropods, the *E*. *fukienensis* mitogenome shows a novel arrangement pattern. Phylogenetic tree supported the monophyly of the superfamily Dermanyssoidea. *Echinolaelaps fukienensis* being the least genetic distant (0.2762) and most closely related to *Varroa destructor*.

**Conclusions:**

This study analyzed comprehensive the structure and evolution of the *E*. *fukienensis* mitogenome for the first time, enriches molecular data of the genus *Echinolaelaps*, which will contribute to further understand phylogeny and rearrangement patterns of the superfamily Dermanyssoidea.

## Introduction

The superfamily Dermanyssoidea is a complex group with rich species diversity in Gamasida, which prefer to parasitic life and their hosts include *Rodentia*, *Insectivora*, *Scandentia*, *Lagomorpha*, *Chiroptera* and small Carnivores [3]. The superfamily Dermanyssoidea is closely related to medicine and important vectors for transmission of epidemic hemorrhagic fever [1–3].

*Echinolaelaps fukienensis* belongs to Arthropoda, Arachnida, Acari, Parasitiformes, Gamasida, Dermanyssoidea and is mainly parasitic on *Niviventer fulvescens*, *Niviventer confucianus*, *Niviventer andersoni* and *Niviventer excelsior* of the genus *Niviventer* [4]. In 1929, Wang first named *E*. *fukienensis* and considered that *E*. *fukienensis* belonged to the genus *Echinolaelaps* [5]. Some acarologists have suggested that *E*. *fukienensis* belonged to the genus *Laelaps* because of characteristics of the genus *Echinolaelaps* were not clearly distinguished from *Laelaps* and renamed *Laelaps fukienensis*, but until now taxonomic system of *E*. *fukienensis*/*L*. *fukienensis* has not formed a consensus [3]. Because *E*. *fukiensis* has a larger body length compared to other mites, the name of *E*. *fukienensis* was used for subsequent analysis in this study.

A typical arthropod mitogenomes contains 37 genes, i.e. 22 tRNA genes (tRNAs), 13 protein-coding genes (PCGs), 2 rRNA genes (rRNAs) and 1 control region of variable length (CR) [6]. Control region is also called non-coding region (NCR) or AT-rich region. The extremely compact structure of mitogenome (16–19 kb), rapid evolutionary rate, and matrilineal inheritance have made it important molecular marker for studying the origin of species, interspecific and intraspecific phylogenetic relationships in recent years. Current published mitogenomes of Gamasida in NCBI revealed that mitogenomes of *Parasitus wandunqingi*, *Parasitus fimetorum*, *Microdiplogynium sp*, *Quadristernoseta cf*. *longigynium*, *Quadristernoseta cf*. *intermedia* retained arrangement pattern of the ancestral arthropod gene order, while mitogenomes of other mite species showed varying degrees of rearrangement [7–10]. In addition, mitogenomes of *Euseius nicholsi* and *Metaseiulus occidentalis* showed multiple gene duplications [7], which is a relatively rare phenomenon in arthropod mitogenomes. At present, there are few studies on mitogenomes of the superfamily Dermanyssoidea in the world, and there is still a gap in the study of mitogenomes of the genus *Echinolaelaps*. Morphological features and mitogenome of *E*. *fukienensis* were studied to provide novel insights into rearrangement pattern and phylogeny of the superfamily Dermanyssoidea in this study.

## Material and methods

### Collection and morphological identification of specimens

*Echinolaelaps fukienensis* (50 individuals) were collected in 2021 from the body surface of *N*.*confucianus* (Rodentia, Muridae) in Yunnan Province, China. Alive host (*N*.*confucianus*) trapped were placed individually in pre-marked white cotton bags and transferred to laboratory for species identification and parasitological check. Mites on the body surface of each host were collected and preserved in 95% ethanol at −80°C prior to DNA extraction. *Echinolaelaps fukienensis* and their host *N*. *confucianus* are preserved at Institute of Pathogens and Vectors, Dali University.

Specimens of *E*. *fukienensis* were taken from anhydrous ethanol solution and placed in a petri dish. It was then rinsed with distilled water and fixed onto a microscope slide using Hoyer’s medium and cover glass. After air-drying, the specimen was photographed for morphological identification under Leica microscope following Deng’s descriptions [3]. The morphology image of *E*. *fukienensis* were edited using Adobe Photoshop.

### DNA extraction, mitogenome sequencing and analysis

Total DNA were extracted from 50 individual mite with DNeasy Tissue kit (QIAGEN) following the manufacture’s protocol and was assayed for concentration and purity using NanoDrop, and was sent to Shanghai Winnerbio Technology Co., Ltd. (Shanghai, China) for sequencing using Illumina Novoseq 6000 platform. About 1 μg DNA was sheared into 400–500 bp fragments using a Covaris M220 Focused Acoustic Shearer following manufacture’s protocol. Illumina sequencing library were prepared from the sheared fragments, followed by paired-end sequencing (2 × 150 bp) on an Illumina NovaSeq 6000 machine. The raw sequencing reads were quality filtered by fastp (version 0.23.0) to obtain 4 Gb of clean data. Illumina clean reads were assembled using Geneious Primer software. The assembly parameters were minimum overlap 50 bp and minimum overlap identity 98%. tRNA genes were identified with tRNAscan-SE and ARWEN [11, 12], protein-coding genes and rRNA genes were identified with Geneious Primer software, BLAST and MITOS [13, 14]. Base composition and codons were analyzed using Geneious Prime and Codon W respectively [15]. Nucleotide composition skew was calculated using the follow formula: AT skew = (A—T) / (A + T) and GC skew = (G—C) / (G + C).

To verify accuracy of sequencing results, *cox1* short fragment nucleic acid sequences of *E*. *fukienensis* known from the NCBI database were traced and verified, we mapped all sequencing reads to partial *cox1* sequences for 1000 iterations using “Map to Reference” function in Geneious Prime and get the same mitogenome sequence.

### Mitogenome arrangement and phylogeny

To visualize gene order between mitochondrial genes of *E*. *fukienensis* and other 6 known species of the superfamily Dermanyssoidea, we made gene arrangement starting with *cox1* for mitogenomes of these species for comparative analysis. Genetic distance of the mitogenomes sequences of 7 species in the superfamily Dermanyssoidea was analyzed using MEGA 11. Meanwhile the Bayesian method (BI method) [16] was used to construct phylogenetic tree based on the complete mitogenomes sequences of 7 species in the superfamily Dermanyssoidea. Optimal model was GTR+I+G model by MrMtgui analysis. In Bayesian analysis, four simultaneous Markov chains were run for 1 million generations and trees were sampled every 100 generations. Phylogenetic relationships of 7 species were inferred from evolutionary tree and genetic distance.

### Ethics statement

*Niviventer confucianus* were handled in strict accordance with good animal practice as defined by relevant national and/or local animal welfare bodies, and animal capture protocols and procedures were approved by Animal Ethics Committees at Dali University (approval # MECDU-201806-11). All collection methods were carried out in accordance with the approved guidelines and regulations.

## Results

### Morphological characterization of *E*. *fukienensis*

**Fig 1:**
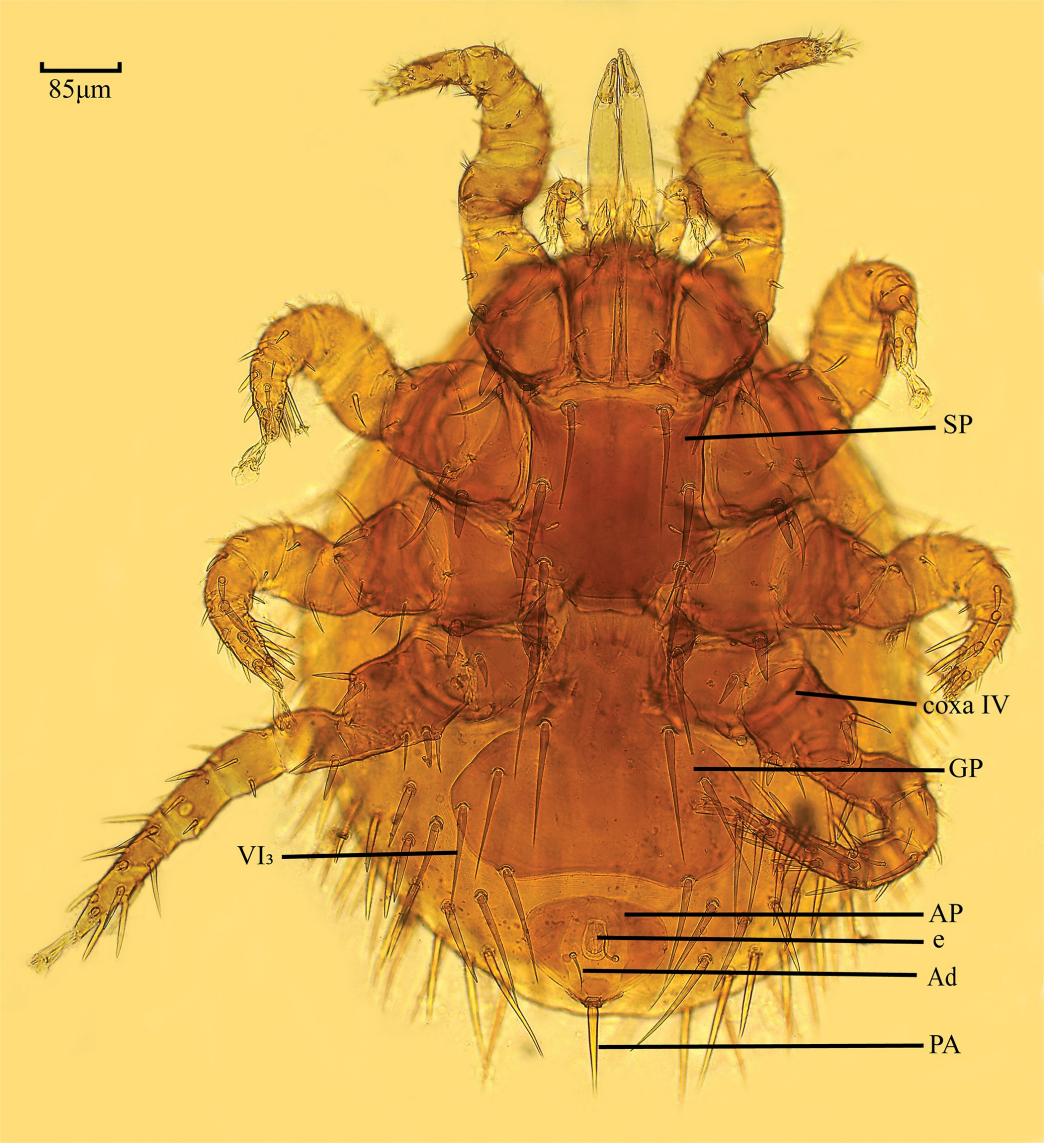
Morphological diagram of *Echinolaelaps fukienensis*. Note: SP stands for sternal shield; coxa IV stands for the fourth pair of coxae; GP stands for genito-ventral shield; VI3 stands for genito-ventral plate 3rd pair of setae; AP stands for anal shield; e stands for anus; Ad stands for adanal stea; PA stands for postanal stea.

Morphological features of *E*. *fukienensis* were shown in the following Fig 1. Morphology of *E*. *fukienensis* is ovoid, several cover the entire dorsum, plate with 39 pairs of needle-like setae, arranged as in the *genus Laelaps*, S8 is 0.065 mm, M11 is 0.159 mm, length of sternal shield is greater than width, genito-ventral shield is expanded after basal coxa IV, genito-ventral shield is widest at VI3, VI4 is located at the posterior margin of genito-ventral shield, the anal shield is fan-shaped, with the middle of the anterior margin is slightly concave inward, adanal stea is smaller located on the transverse line of the posterior margin of the anus, postanal stea is coarse. Each leg coxa are with one coarse spiny seta, coxa IV is shorter, dorsal surface of femur of foot I has a pair of long setae [5].

### Mitogenome structure of *E*. *fukienensis*

**Fig 2:**
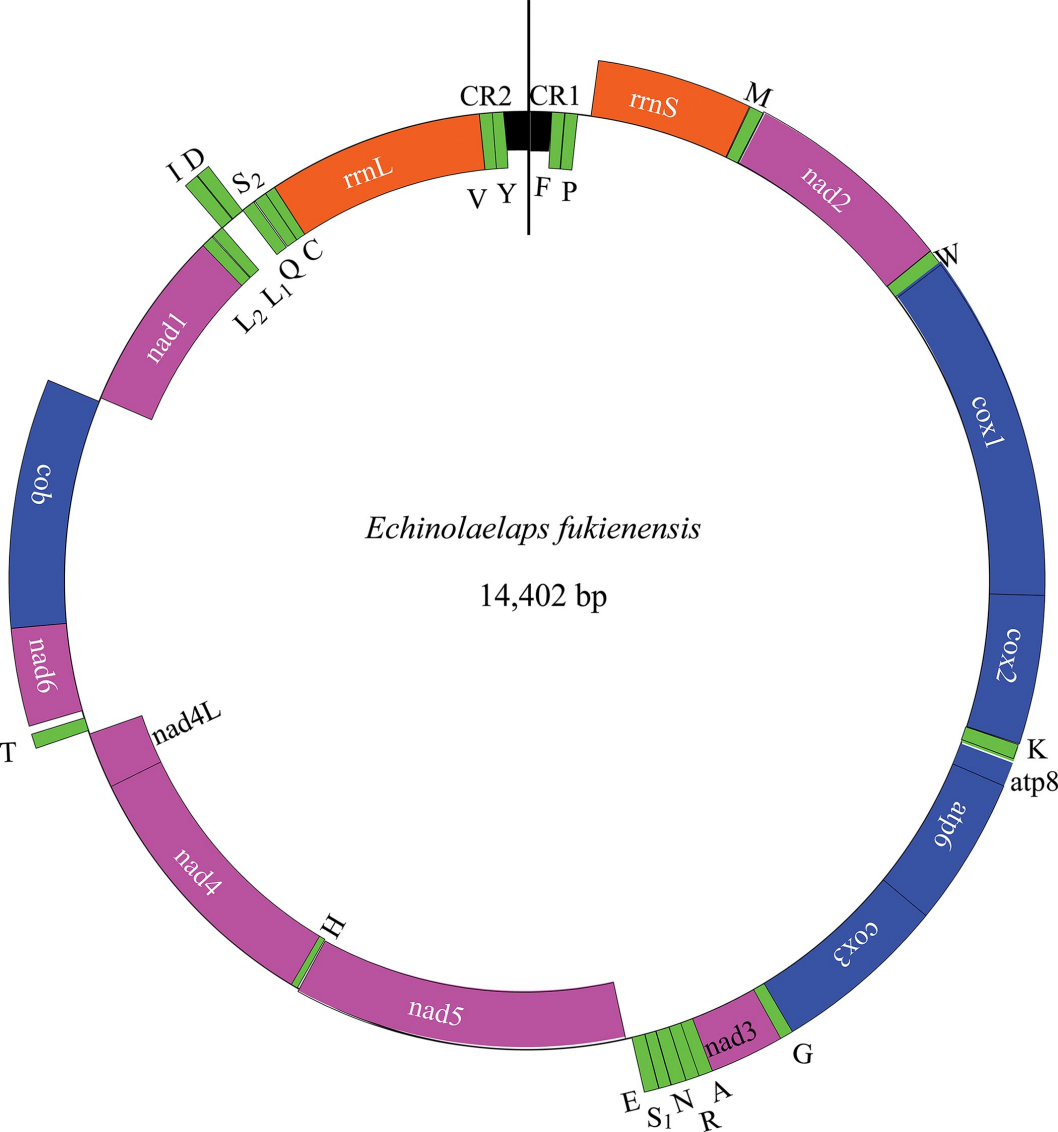
The complete mitogenome of *Echinolaelaps fukienensis*. Genes on the outside of the circle are coded on the major or J strand, whereas genes on the inside of the circle are on the complement (minor or N) strand.

The *E*. *fukienensis* mitogenome is a typical closed-loop double-stranded DNA molecule, encoding 37 genes, including 13 protein-coding genes (PCGs), 22 tRNA genes, 2 rRNA genes and 1 control region, with a size of 14,402 bp, which is known the smallest genome of the superfamily Dermanyssoidea and has a high AT content (81.4%). There are genes on both strands of mitogenome, with 22 genes on the heavy strand, including 9 protein-coding genes (*nad2*, *cox1*, *cox2*, *atp8*, *atp6*, *cox3*, *nad3*, *nad6 and cob*), 12 tRNA genes (*trnM*, *trnW*, *trnK*, *trnG*, *trnA*, *trnR*, *trnN*, *trnS*_*1*_, *trnE*, *trnT*, *trnI and trnD*), and 1 rRNA gene (*rrnS*). The remaining 15 genes (*trnF*, *trnP*, *nad5*, *trnH*, *nad4*, *nad4L*, *nad1*, *trnL*_*2*_, *trnL*_*1*_, *trnQ*, *trnS*_*2*_, *trnC*, *rrnL*, *trnV*, *trnY*) are on the light strand (Genbank accession number: OQ603510 Fig 2). The *E*. *fukienensis* mitogenome has 1 control region and and the size is 229 bp (see Table 1).

**Table 1:** Organization of the *Echinolaelaps fukienensis* mitogenome.

Genes	Strand	Position	Length	AT-skew	GC-skew	Start codon	Stop codon	Anticodon
*CR1*		1–122	122					
*trnF*	N	123–186	64					GAA
*trnP*	N	189–252	64					TGG
*rrnS*	J	318–1018	701					
*trnM*	J	1022–1087	66					CAT
*nad2*	J	1088–2047	960	-0.118	0.244	ATA	TAA	
*trnW*	J	2046–2111	66					TCA
*cox1*	J	2112–3665	1554	-0.183	0.142	ATC	TAA	
*cox2*	J	3665–4340	676	-0.105	0.098	ATG	T-tRNA	
*trnK*	J	4341–4406	66					CTT
*atp8*	J	4414–4521	108	0	0	ATT	TAA	
*atp6*	J	4521–5186	666	-0.145	0.138	ATG	TAA	
*cox3*	J	5186–5968	773	-0.201	0.254	ATG	TAA	
*trnG*	J	5968–6030	63					TCC
*nad3*	J	6031–6363	333	-0.322	0.32	ATT	TAG	
*trnA*	J	6362–6425	64					TGC
*trnR*	J	6425–6485	61					TCG
*trnN*	J	6486–6553	68					GTT
*trnS*_*1*_	J	6555–6610	56					TCT
*trnE*	J	6612–6677	66					TTC
*nad5*	N	6709–8391	1683	0.143	0.191	ATT	TAA	
*trnH*	N	8392–8457	66					GTG
*nad4*	N	8458–9760	1303	0.176	0.097	ATG	T-tRNA	
*nad4L*	N	9760–10038	279	0.243	0.045	ATT	TAA	
*trnT*	J	10057–10121	65					TGT
*nad6*	J	10158–10592	435	-0.172	0.235	ATT	TAA	
*cob*	J	10593–11699	1107	-0.222	0.199	ATG	TAA	
*nad1*	N	11719–12624	906	0.162	-0.031	ATA	TAA	
*trnL*_*2*_	N	12625–12687	63					TAA
*trnL*_*1*_	N	12692–12754	63					TAG
*trnI*	J	12752–12818	67					GAT
*trnD*	J	12822–12884	63					GTC
*trnQ*	N	12887–12954	68					TTG
*trnS*_*2*_	N	12962–13025	64					TGA
*trnC*	N	13027–13079	53					GCA
*rrnL*	N	13080–14165	1086					
*trnV*	N	14166–14230	65					TAC
*trnY*	N	14230–14295	66					GTA
*CR2*		14296–14402	106					

Note: The locations of genes on heavy (J) or light (N)

### Protein-coding genes and codon usage

Nine protein-coding genes (*nad2*, *cox1*, *cox2*, *atp8*, *atp6*, *cox3*, *nad3*, *nad6*, *cob*) are on the heavy strand, and the remaining 4 (*nad5*, *nad4*, *nad4L*, *nad1*) are on the light strand. The total length of 13 PCGs is 10,793 bp, accounting for approximately 74.9% of mitogenome (14,402 bp), AT content of 13 PCGs is 80.7%, and PCGs with the lowest AT content is *cox1* (74.2%) and the highest is *cob* (88%). The longest sequence among 13 protein-coding genes is *nad5* (1683 bp) and the shortest is *atp8* (108 bp). The start codon of all 13 protein-coding genes starts with ATN, 11 protein-coding genes use TAN as the stop codon, while the other 2 protein-coding genes, *cox2* and *nad4*, use the incomplete codon T as the stop codon.

The relative synonymous codon usage (RSCU) of 13 PCGs was shown in Table 2. Codon W is used to analyze codon usage corresponding to tRNA genes in the *E*. *fukienensis* mitogenome, and a total of 3596 codons were analyzed, and Table 2 shows that UUA (421), UUU (409), AUU (396), and AUA (306) were used most frequently, accounting for 42.6% of all codons used. Use of stop codons was relatively normal, and 2 stop codons UAA and UAG played a normal role in termination of mitogenome. In addition, Leucine (Leu), Phenylalanine (Phe), Isoleucine (Ile) and Serine (Ser) as the main amino acids composing proteins also reflect to some extent that codon has an AT base preference.

**Table 2:** Codon usage for 13 PCGs of the *Echinolaelaps fukienensis* mitogenomes.

AA	Codon	n	%	RSCU	AA	Codon	n	%	RSCU
stop	UAA	10	0.28	1.82	Asn(N)	AAU	206	5.73	1.91
	UAG	1	0.03	0.18		AAC	10	0.28	0.09
Ala(A)	GCU	65	1.81	2.86	Pro(P)	CCU	80	2.22	3.02
	GCC	3	0.08	0.13		CCC	4	0.11	0.15
	GCA	23	0.64	1.01		CCA	22	0.61	0.83
	GCG	0	0.00	0.00		CCG	0	0.00	0.00
Cys(C)	UGU	36	1.00	1.85	Gln(Q)	CAA	38	1.06	1.73
	UGC	3	0.08	0.15		CAG	6	0.17	0.27
Asp(D)	GAU	57	1.59	1.90	Arg(R)	CGU	20	0.56	1.78
	GAC	3	0.08	0.10		CGC	0	0.00	0.00
Glu(E)	GAA	77	2.14	1.73		CGA	24	0.67	2.13
	GAG	12	0.33	0.27		CGG	1	0.03	0.09
Phe(F)	UUU	409	11.37	1.92	Ser1(S_1_)	AGU	25	0.70	0.58
	UUC	16	0.44	0.08		AGC	6	0.17	0.14
Gly(G)	GGU	67	1.86	1.72		AGA	110	3.06	2.54
	GGC	4	0.11	0.10		AGG	11	0.31	0.25
	GGA	69	1.92	1.77	Ser2(S_2_)	UCU	104	2.89	2.40
	GGG	16	0.44	0.41		UCC	6	0.17	0.14
His(H)	CAU	66	1.84	1.89		UCA	80	2.22	1.85
	CAC	4	0.11	0.11		UCG	4	0.11	0.09
Ile(I)	AUU	396	11.01	1.89	Thr(T)	ACU	64	1.78	2.21
	AUC	24	0.67	0.11		ACC	0	0.00	0.00
Lys(K)	AAA	105	2.92	1.89		ACA	51	1.42	1.76
	AAG	6	0.17	0.11		ACG	1	0.03	0.03
Leu1(L_1_)	CUU	50	1.39	0.57	Val(V)	GUU	65	1.81	1.68
	CUC	4	0.11	0.05		GUC	3	0.08	0.08
	CUA	32	0.89	0.37		GUA	77	2.14	1.99
	CUG	2	0.06	0.02		GUG	10	0.28	0.26
Leu2(L_2_)	UUA	421	11.71	4.81	Trp(W)	UGA	68	1.89	1.84
	UUG	16	0.44	0.18		UGG	6	0.17	0.16
Met(M)	AUA	306	8.51	1.91	Tyr(Y)	UAU	161	4.48	1.82
	AUG	14	0.39	0.09		UAC	16	0.44	0.18

RSCU: Relative synonymous codon usage.

### rRNA and tRNA genes

As most animals mitogenomes, the *E*. *fukienensis* mitogenome has 2 rRNA genes. *RrnS* is on the heavy strand, 701 bp in size, and *rrnL* is on the light strand, 1086 bp in size (Table 1 and Fig 1). *RrnS* is between *trnP* and *trnM*, *rrnL* is between *trnC* and *trnV*, both rRNAs are close to control region but not connected, and the interval is the distance of 2 tRNAs. Nucleotide composition of rRNA showed a strong AT bias, with 81.9% and 83.8% AT content in *rrnS* and *rrnL*, respectively.

**Fig 3:**
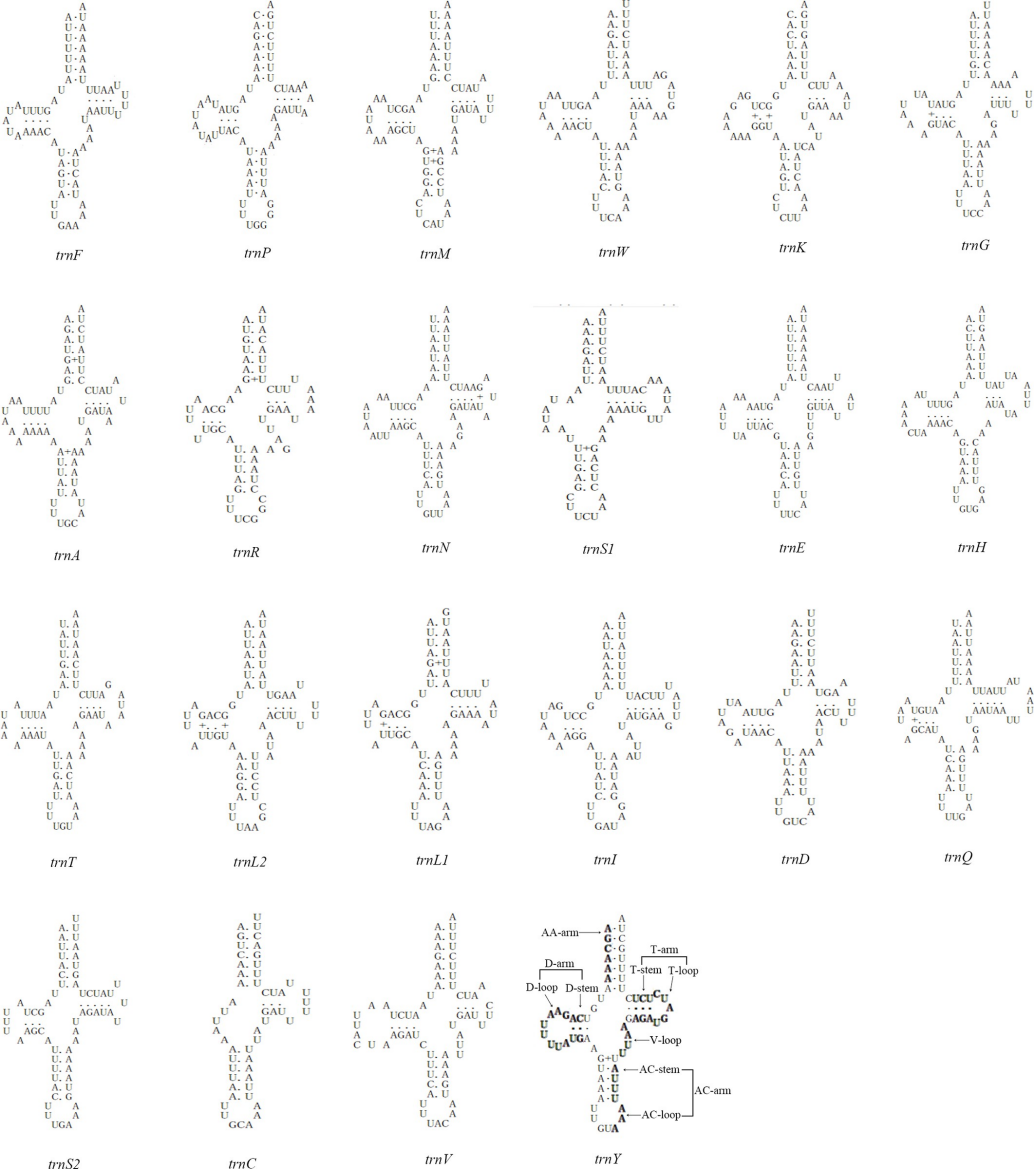
Putative secondary structures of the 22 mitochondrial tRNA genes of *Echinolaelaps fukienensis*.

The *E*. *fukienensis* mitogenome has 22 tRNA genes, of which 12 tRNA genes are on the J-strand and the remaining 10 tRNA genes are on the N-strand, see Table 1 and Fig 2. The size of these tRNAs range from 53 bp (*trnC*) to 68 bp (*trnQ* and *trnN*), with an average length of 64 bp, slightly smaller than the size of arthropod tRNA genes (66 bp). Most tRNAs secondary structure have regular clover-leaf secondary structures, except for *trnC* and *trnS*_*1*_ lacking the D-arm, see Fig 3. Predicted tRNAs secondary structure showed base mismatches and use of unconventional codons in some tRNA genes, with *trnM* showing an A-G mismatch, *trnM*, *trnG*, *trnR*, *trnS*_*1*_, *trnN*, *trnQ*, *trnA* and *trnY* all showing 1 G-U mismatch; *trnK*, *trnL*_*2*_ and *trnL*_*1*_ all showing 2 G-U mismatches. In addition, *trnK* uses the unconventional anticodon CUU (CUU instead of the standard UUU), which was found for the first time in the superfamily Dermanyssoidea. Predicted tRNA secondary structure had fully paired amino acid acceptor stem, but in anticodon loop, *trnS*_*2*_ and *trnC* appear lengthened by 1 bp each, and there are missing bases between the acceptor stem and D-arms in *trnC*, which may be a unique feature of the *E*. *fukienensis* mitogenome.

### Control region and its stem-loop structure

**Fig 4:**
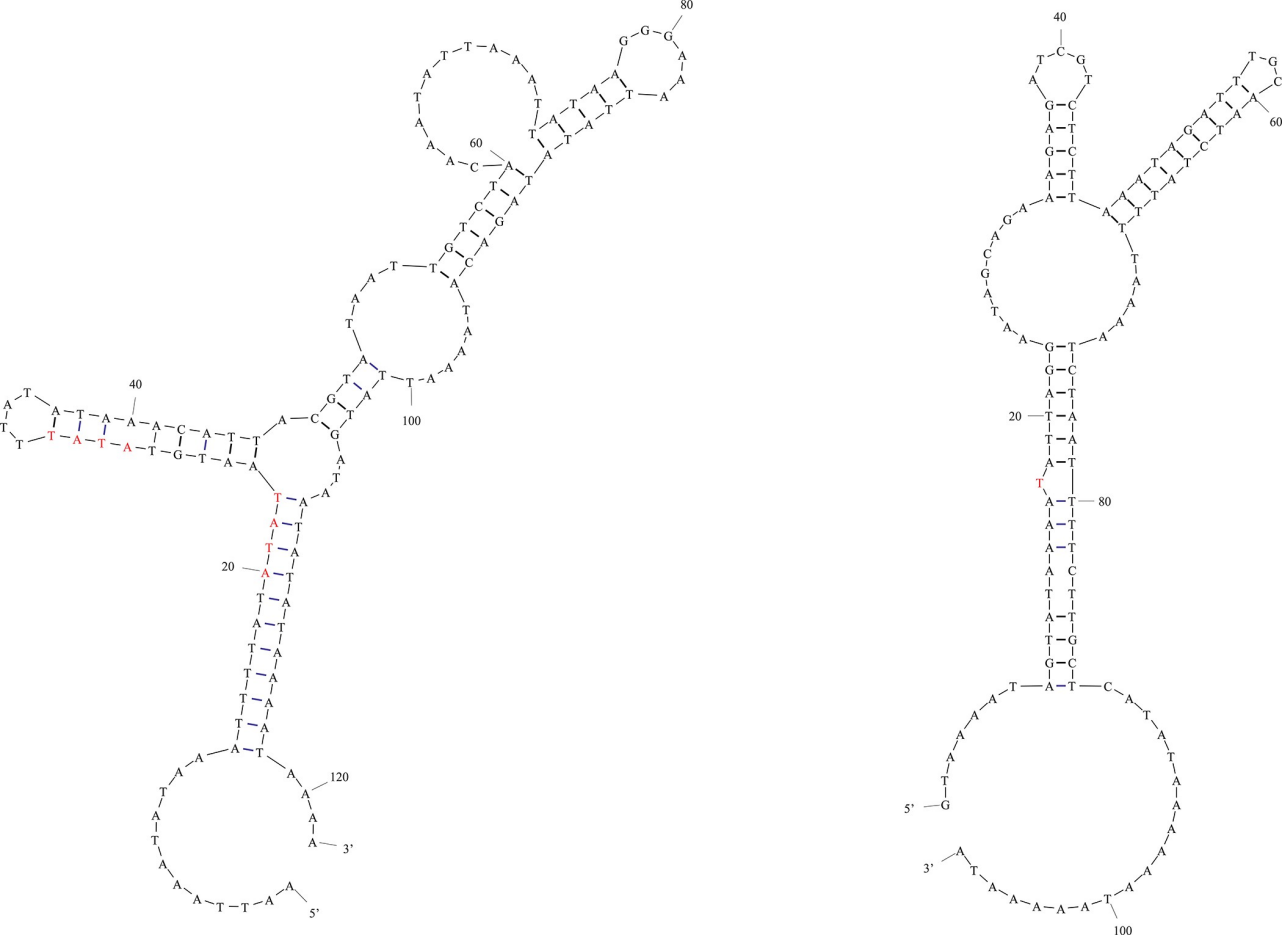
Secondary structures of control region in the *Echinolaelaps fukienensis* mitogenome.

There is 1 control region (>50 bp) in the *E*. *fukienensis* mitogenome, located between *trnF* and *trnY*, which contain abundant AT content, reaching 84.7%. To better analyze the nucleotide composition of control region of *E*. *fukienensis*, control region of *E*. *fukienensis* was visualized and analyzed. There are multiple stem-loop structures by visual analysis of control region structure in *E*. *fukienensis* (see Table 3 and Fig 4). Structure microsatellite-like (AT) n was found in control region. Structure microsatellite-like (AT) n was present in 2 control regions of *Hypoaspis linteyini* and *Coleolaelaps cf*. *liui*, which also belonging to the family Laelapidae. Structure microsatellite-like (AT) n may be synapomorphy of the family Laelapidae. Meanwhile, multiple palindromic sequences were found in control regions. Palindromic sequences refer to sequences read from the 5’ end of a sequence or from the 5’ end of its complementary strand are the same, and palindromic sequences have mirror symmetry in DNA or RNA. Sequence size is set for online sites that filter palindrome structure. The length of filtered sequence varies in size, but generally palindrome sequence length is not very long.

**Table 3:** Palindromic sequence in control region of *Echinolaelaps fukienensis*.

Control region					
Position	Length	Sequence	Position	Length	Sequence
11	6	AAATTT	14305	4	TATA
16	10	TTATATATAA	14310	6	AATATT
17	8	TATATATA	14311	4	ATAT
18	6	ATATAT	14332	4	GATC
19	6	TATATA	14341	4	TTAA
32	10	TTTATATAAA	14351	6	TTGCAA
33	8	TTATATAA	14352	4	TGCA
34	6	TATATA	14360	10	ATTTTAAAAT
45	6	TACGTA	14361	8	TTTTAAAA
63	6	AATATT	14362	6	TTTAAA
72	6	TTATAA	14363	4	TTAA
85	6	TATATA	14372	4	AATT
108	6	ATATAT	14385	4	ATAT
109	6	TATATA	14386	4	TATA

### Mitochondrial gene arrangement patterns

**Fig 5:**
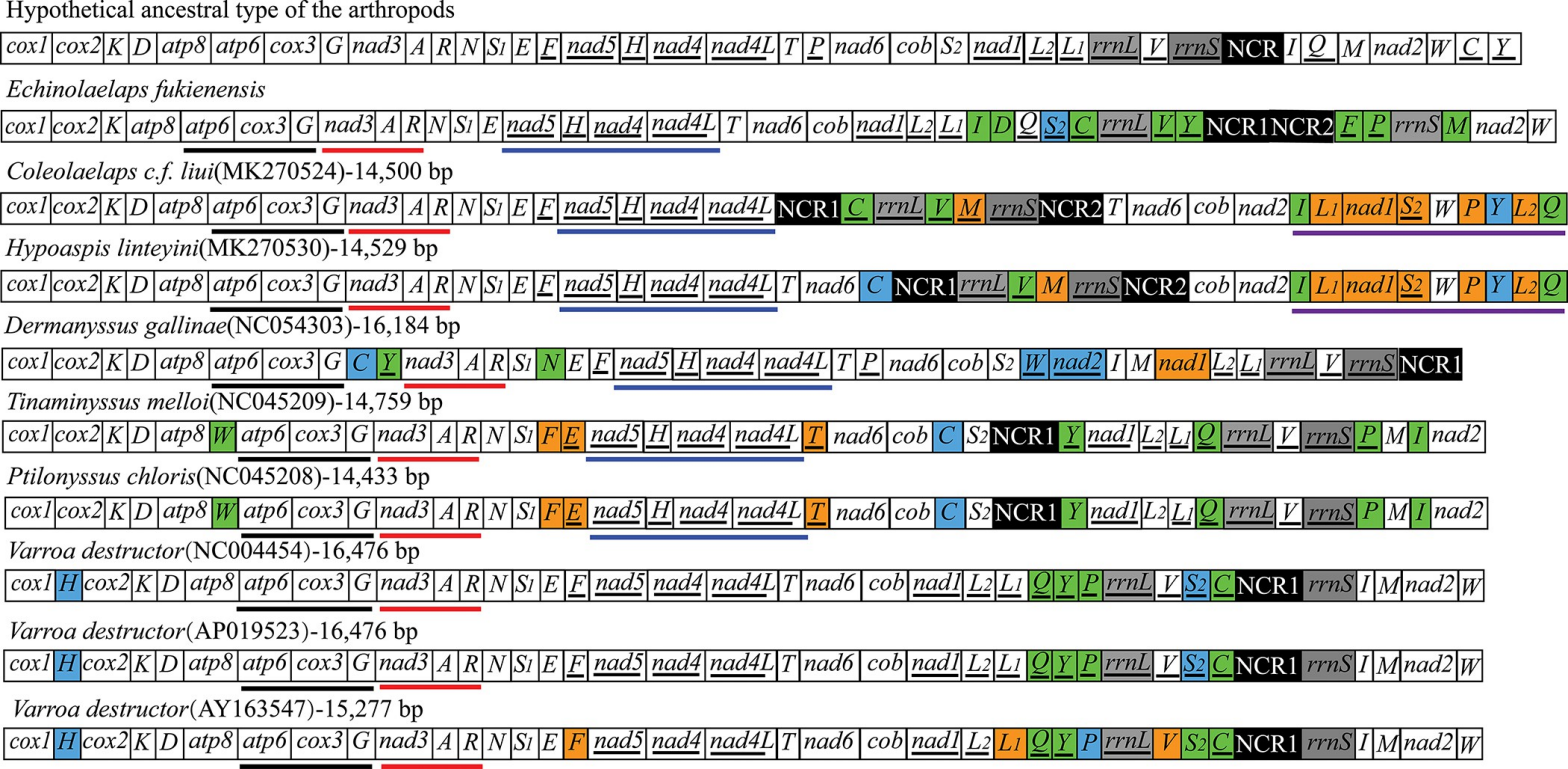
Gene rearrangements of the superfamily Dermanyssoidea mitogenomes. Translocated or inverted genes are colour-coded (blue: inversion and translocation; green: translocation; orange: inversion) except *rrnL* and *rrnS* genes (grey in colour); non-coding regions (NCR) are in black. The colored lines represent shared gene clusters between or among species. (For interpretation of the references to colour in this figure legend, the reader is referred to the web version of this article).

Gene arrangement has been used as a phylogenetic feature for many lineages, including mesostigmatid mites of previous studies. Gene arrangements were mapped based on gene positions in the superfamily Dermanyssoidea mitogenomes to explore gene arrangements variation among them. Gene arrangements of 3 *V*. *destructor* mitogenomes are mapped for comparison in Fig 5. The *E*. *fukienensis* mitogenome showed a unique gene arrangement pattern when compared to hypothetical ancestral arthropod mitogenome gene order. Mitogenome gene arrangement patterns have variation in different species of the superfamily Dermanyssoidea. Fig 5 showed that *trnI*, *trnD*, *trnC*, *trnV*, *trnY*, *trnF*, *trnP* and *trnM* of *E*. *fukienensis* have undergone translocation, and *trnS*_*2*_ has undergone transposition and inversion. Compared to genes that have undergone translocation and transposition inversion, most genes of *E*. *fukienensis* are conserved state. Genes of the other 6 species of Dermanyssoidea are also mostly conserved and shared *atp6*-*cox3*-*G* and *nad3*-*A*-*R* gene clusters, with difference that *rrnS* gene of *E*. *fukienensis* and *V*. *destructor* are on the light strand. In terms of the number of mitochondrial genes, only *Dermanyssus gallinae* had 36 genes with missing *trnQ*. Fig 5 showed that *H*. *linteyini* and *C*. *cf*. *liui* as the family Laelapidae shared more gene clusters. *Ptilonyssus chloris* and *Tinaminyssus melloi* showed the same pattern of mitochondrial gene arrangement but size difference. Other 6 species of Dermanyssoidea shared *nad5*-*H*-*nad4*-*nad4L* gene cluster*s* except *V*. *destructor*. Comparison with mitogenome gene order of 3 *V*. *destructor* (AY163547 from America), *V*. *destructor* (NC004454 from France)) and *V*. *destructor* (AP019523 from Japan) have the same genome size and the same gene arrangement pattern but gene content have difference, whereas *V*. *destructor* (AY163547 from America) has smaller mitogenome size and gene arrangement pattern difference than the other 2 *V*. *destructor*.

### Phylogenetic analysis

**Fig 6:**
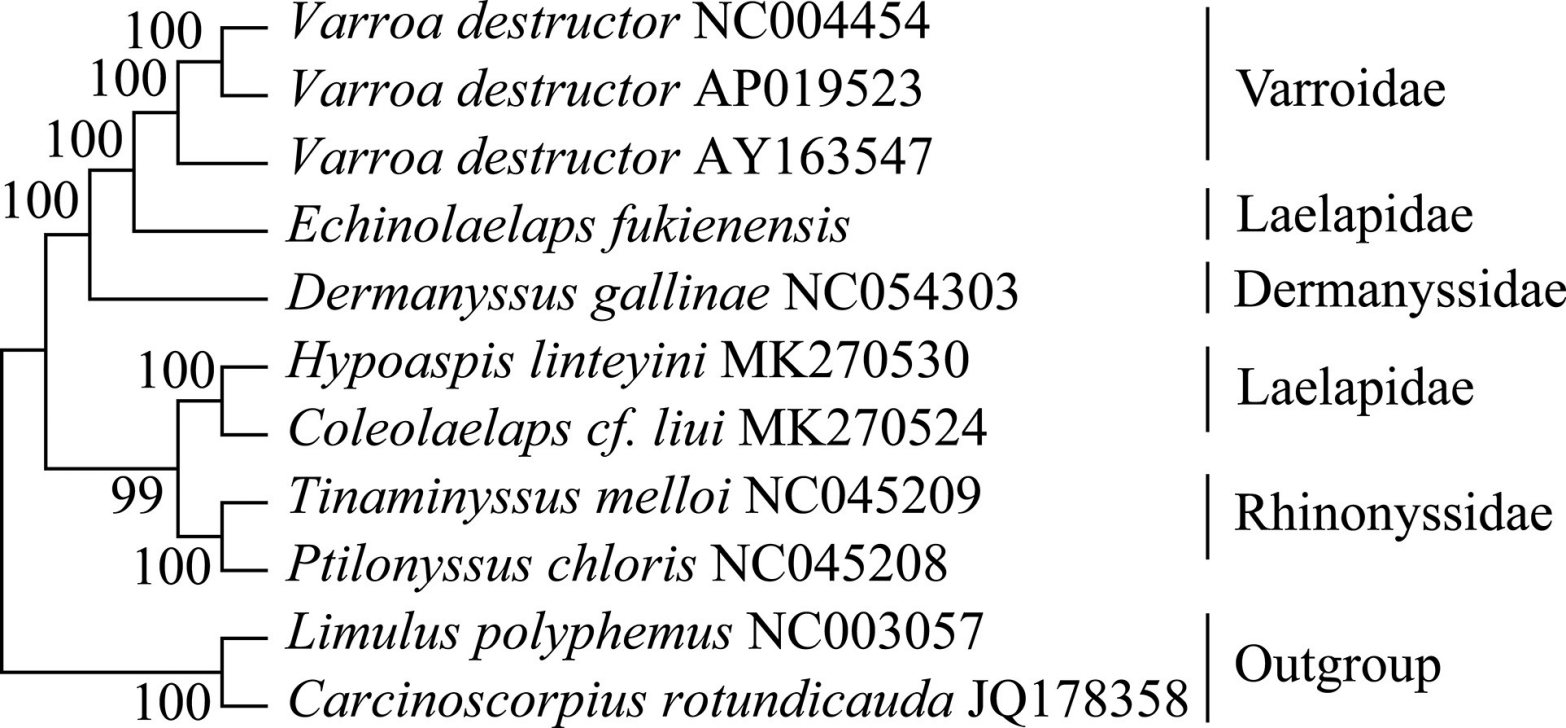
Phylogenetic tree of the superfamily Dermanyssoidea based on mitogenome sequences.

Phylogenetic tree was constructed based on the mitogenome sequences of 7 species in the superfamily Dermanyssoidea, of which *V*. *destructor* belongs to the family Varroidae; *E*. *fukienensis*, *H*. *linteyini* and *C*. *cf*. *liui* belong to the family Laelapidae; *D*. *gallinae* belongs to the family Dermanyssidae; *P*. *chloris* and *T*. *melloi* belong to the family Rhinonyssidae; *Carcinoscorpius rotundicauda* and *Limulus polyphemus* were used as outgroups. Phylogenetic analysis supports the monophyly of the superfamily Dermanyssoidea (Fig 6). Mitogenome data of *V*. *destructor* included in this study come from 3 countries, *V*. *destructor* (NC004454 from France), *V*. *destructor* (AY163547 from America), *V*. *destructor* (AP019523 from Japan). *V*. *destructor* with different geographical regions have differences in their mitogenomes size, base content, gene order and genetic distance (0.0232), but differences are small. Phylogenetic tree showed that *V*. *destructor* (France) and *V*. *destructor* (Japan) were clustered together, and genetic distance is close to 0.0014 between them. *H*. *linteyini* and *C*. *cf*. *liui* from the family Laelapidae were clustered together, while *E*. *fukienensis*, which is a member of the family Laelapidae, forming a sister group with *V*. *destructor*, and showing a close relationship. MEGA 11 was used to analyze genetic distance between mitogenomes of the superfamily Dermanyssoidea (see Table 4). Genetic distance between *E*. *fukienensis* and *V*. *destructor* (Japan) of the family Varroidae was the closest (0.2762), and that between *E*. *fukienensis* and *C*. *cf*. *liu* from the family Laelapidae is the farthest (0.4538).

**Table 4:** Genetic distance among mites of the superfamily Dermanyssoidea based on mitogenomes sequences.

	1	2	3	4	5	6	7	8	9
1.*Echinolaelaps fukienensis*									
2.*Hypoaspis linteyini* MK270530	0.4131								
3.*Coleolaelaps cf*. *liui* MK270524	0.4538	0.3471							
4.*Dermanyssus gallinae* NC054303	0.3756	0.4624	0.4844						
5.*Tinaminyssus melloi* NC045209	0.3960	0.4150	0.4329	0.4021					
6.*Ptilonyssus chloris* NC045208	0.3549	0.3959	0.4304	0.3669	0.3210				
7.*Varroa destructor* NC004454	0.2768	0.4499	**0.4901**	0.3918	0.4068	0.3622			
8.*Varroa destructor* AY163547	0.2769	0.4439	0.4880	0.3922	0.4067	0.3627	0.0242		
9.*Varroa destructor* AP019523	**0.2762**	0.4492	0.4893	0.3911	0.4060	0.3614	**0.0014**	**0.0232**	

Note: The numbers of genetic distance are shown below the diagonal.

## Discussion

The *E*. *fukienensis* mitogenome was obtained for the first time in this study with 37 genes of typical arthropod mitogenomes and 14,402 bp in size, which is known the smallest genome of the superfamily Dermanyssoidea. By comparing mitogenomes of *H*. *linteyini* and *C*. *cf*. *liui* of the same family, variation in mitogenomes size is mainly reflected in control region, and the length of control region of *E*. *fukienensis* is smaller than that of *H*. *linteyini* and *C*. *cf*. *liui*. It is reported that in the process of animal evolution, there is a tendency for mitogenomes to decrease in size, and even during the reduction process, gene loss may occur [17, 18]. Furthermore, genetic studies have shown that smaller mitogenomes are more advantageous than larger ones in the process of transmission to offspring, implying that natural selection tends to select smaller mitogenomes during evolution. Mitochondria play an important role in cellular energy metabolism, and metabolic differences may affect the evolution of mitogenomes, species with higher metabolic rates tend to have smaller mitogenomes [19]. *Echinolaelaps fukienensis* may have a higher metabolic rate, and theoretically, its mitogenome size is more stable. With the passage of time and environment, the changes in size and internal structure of *E*. *fukienensis* mitogenome may indicate that harmful mutations have been overcome, causing mitogenome to evolve towards smaller size to reduce their own material consumption [18, 19]. Normally, rRNAs genes (*rrnS* and *rrnL*) of arthropod mitogenomes are on the N-strand and near the largest control region [20], while *rrnS* of *E*. *fukienensis* is on the J-strand. The *E*. *fukienensis* mitogenome had rearrangement. *Nad5* has the longest length and *atp8* has the shortest length among 13 PCGs, which may be synapomorphies of animal mitogenomes. Surprisingly, base composition of *atp8* is A = T and C = G, i.e. AT-skew and GC-skew of *atp8* are both 0. Similar situation had not been seen in mites. There also been special situations in codons usage of the *E*. *fukienensis* mitogenome. In general, all PCGs began with ATN (ATG, ATA and ATT) as the start codon, except *cox1* gene with ATC as the start codon, which is rare in mites, however, it also appeared in *Neoseiulus womersleyi* and *Amphitetranychus viennensis* mitogenomes [10, 21]. The stop codon of most protein-coding genes of *E*. *fukienensis* mitogenome is TAA, and only the stop codon of *nad3* is TAG, while *cox2* and *nad4* have incomplete codon T as the stop codon, and the occurrence of incomplete stop codons is common in mites [10], such as the occurrence of TAG and ATA in the stop codon of *Euseius nicholsi* [22], the occurrence of incomplete T and TA in the stop codon of *Psoroptes cuniculi* [23], and the occurrence of T and TAG in the stop codon of *H*. *linteyini*, which is a member from the superfamily Dermanyssoidea, phenomenon of incomplete stop codons in mites is believed to be completed through post-transcriptional polyadenylation to form TAA to exercise the function of termination codons [20, 24]. Usually stop codon TAA or TAG can always overlap several nucleotides within downstream tRNA. This situation occurred in Megaloptera as a "backup" to prevent translation read through, and also affects some amino acids usage [24], however, part stop codon of 13 protein-coding genes in the *E*. *fukienensis* mitogenome did not overlap with tRNA. This may be relatively small size of mites and short generation times, which led to the high evolutionary rate, and different degrees of gene variation occurred at the mitogenome level.

Most tRNAs secondary structures are typical clover-leaf structures, with only *trnC* and *trnS*_*1*_ lacking the D-arm, *trnS*_*1*_ missing the D-arm is a typical feature of metazoan mitogenomes [24], *trnC* missing the D-arm is relatively rare, among which *Centruroides limpidus*, *Nymphon gracile*, and *Achelia bituberculata* have *trnC* missing the D-arm [25], but missing the D-arm or T-arm did not affect its function [26], which is a common occurrence in mites [10, 27–29]. Truncated tRNAs are related to EF-TU proteins in the nematodes mitogenomes [29]. EF-TU1 recognizes tRNAs with missing T-arm, while EF-TU2 recognizes tRNAs with missing D-arm. While the presence of EF-TU genes is essential for nematodes, for how does EF-TU affect the secondary structure of tRNAs? One explanation is that in ancestral species a original single EF-TU gene was duplicated and subsequently co-evolved with the respective discrete mitochondrial tRNAs which may have diverged as genome length decreased, with the final result being a truncated tRNA [30, 31]. How does truncated tRNA function? There is no clear explanation in arthropod, and there are reports that nuclear tRNAs are recruited into mitochondria [32], which makes it unknown whether the pre-existing truncated tRNAs are functional. However, Juhling et al. demonstrated through computational analysis that mitochondrial tRNAs lacking both the D and T arms are functional in nematodes [31, 33]. The occurrence of truncated tRNAs in arthropod mitochondria may be beneficial to species evolution, and these truncated tRNAs may also be functionally involved in the transmission of genetic information, and their occurrence in *E*. *fukienensis* indicates that species are constantly undergoing natural selection. In addition, a total of 15 mismatches occurred in 22 tRNA genes of the *E*. *fukienensis* mitogenome, for which base mismatches in tRNA structure are frequent in other mites, such as acrid mite [34], and base mismatches in stem-loop in tRNA secondary structures of both Arachnid and velvet worms [35, 36], and G-U mismatches are important for maintaining the secondary structure of tRNAs, perhaps species evolution has tacitly accepted that G-U pairing is a normal occurrence, other base mismatches that appear in tRNA secondary structure may be an adaptation chosen by mites to adapt to natural selection during the evolutionary process, as this type of base mismatch is widespread in Acarina [25, 27]. It seems that point mutation exist in the genes of these small animals to constantly adapt to natural selection, although to a lesser extent, it may be a better adaptation process for them. This type of base mismatch can be corrected through editing and will not affect the transport of amino acids, which means that the translation process will not be affected by tRNA base mismatches during protein synthesis [27, 37]. Perhaps for mites in a constantly changing natural environment, synthesizing corresponding functional genes to meet the needs of life activities under the premise of reducing their own material consumption is an ongoing evolution. Anticodon loop has 7 base pairs in tRNA secondary structure, whereas *trnA* of the *E*. *fukienensis* mitogenome had only 6 pairs, while *trnS*_*2*_ and *trnC* had 8 pairs, similar situation also occured in *trnS*_*1*_ and *trnY* of *Psoroptes cuniculi*, *trnS*_*1*_ and *trnK* of *Phytoseiulus persimilis*, and *trnA* and *trnV* in *Paraleius leontonychus* [20, 23, 26], possibly indicating that base pair number of anticodon loop of tRNAs in mites is not very stable and that this occurrence in mites may be the result of their continued evolution.

Normally, TATA sequences of palindromic sequences have different variance but TATA content is constant, and itself is short palindromic structure and consensus core sequence [38]. There are multiple TATA sequences in control region of *E*. *fukienensis*, indicating a high trend of variation in control region, and there is a mismatched base T in the stem of control region (marked in red in Fig 4). Correlational studies had shown that control region forms even if every 6 base pairs were mismatched. It is possible that certain key proteins or repair factors exist in *E*. *fukienensis* to ignore this simple error and synthesize control regions [39], or it may be that the mitogenomes structure of small mites is continued evolution because mismatched base T was also found in control region of *M*. *occidentalis* [40]. Curiously, palindromic sequences 5’TACAT and 3’ATGTA were not found in control regions of *E*. *fukienensis* mitogenome, but this occurred in control regions of *Carpoglyphus lactis* and *Tyrophagus longior* [34, 41]. Usually palindromic sequences TACAT and ATGTA probably function as recognition sites for the arrest of J-stand synthesis in mammals and fish [34], but their unstable presence in small mites may represent the occurrence of higher convergent evolution in mites. Palindromic sequences were found in control regions are not very long and may be recognition sequences of restriction enzyme [42–44]. It has been previously suggested that sequence flanking stem-loop structure is highly conserved among arthropods, with 5’TATA and 3’GA(A)T motif, which are presumed to play important role in replication and transcription of mitogenome. 5’TATA motif was found in control region (marked with red bases), while GA(A)T was not found in control regions of *E*. *fukienensis* mitogenome, and these 2 motifs were also not found in *Panonychus citri* [45, 46]. Usually, control region of animal mitogenomes have highly variable, and the extension sequence poly-T is relatively conserved, poly-T (>10 bp) is indispensable for replication origin of mitogenome, and no extension sequence poly-T (>10 bp) was found in control region of the *E*. *fukienensis* mitogenome. It is possible that other sequences replaced T extension to provide very important information for replication origin of the *E*. *fukienensis* mitogenome without the long fragment T extension.

*Echinolaelaps fukienensis* belongs to the superfamily Dermanyssoidea, and arrangement pattern of mitogenome is still variation with that of other species in Dermanyssoidea. 13 protein-coding genes retain the ancestral arthropods gene order, while tRNA and rRNA showed varying degrees of rearrangement (Fig 5). Studying gene rearrangements of the *E*. *fukienensis* mitogenome will provide references for evolution of Acari. We show that frequent rearrangements of tRNA genes in gene arrangement pattern map of the superfamily Dermanyssoidea, which is consistent with the previous research result that tRNA genes are more prone to movement than other genes [28, 47]. There is a theory that frequent gene rearrangements involving tRNA may be ascribed to the smaller size of tRNA and an unknown mechanism that makes tRNA more mobile [28]. Although mechanism of gene rearrangement is unclear, gene rearrangements are important for species evolution. Generally speaking, species within the same family have similar or identical gene arrangement orders. It is easy to classify species into different taxa based on gene rearrangements, such as *P*. *chloris* and *T*. *melloi* in the family Rhinonyssidae [8], *Phyllocoptes taishanensis* and *Epitrimerus sabinae* in the family Eriophyoidea [31]. We can infer genetic relationship between different taxonomic category by gene arrangement pattern, which indicates that high-level gene rearrangements may be a typical feature for specific taxon, and species is in continuous evolution [10]. It is surprising that the *V*. *destructor* mitogenome from different countries has variation in size. The sequences of the *V*. *destructor* mitogenome from Japan and France had 99.8% similarity, genome size and gene arrangement pattern is the same. While the *V*. *destructor* mitogenome from America are 1199 bp smaller than both from Japan and France and have variation in gene arrangements. As for variation in genome size of the same species, is it the result of geographical differences, climate reasons, etc., or were the data sequenced and annotated from America wrong? This needs further study. We suggest collecting the same species from different regions or different hosts for analysis in the future. Genetic distances and phylogenetic trees revealed that *H*. *linteyini* and *C*. *cf*. *liui* are members of the superfamily Dermanyssoidea, clustered together as the same family Laelapidae, while *H*. *linteyini* and *C*. *cf*. *liui*, which also are members of the superfamily Dermanyssoidea, had genetic distances greater than that of *E*. *fukienensis* and the family Rhinonyssidae. *Echinolaelaps fukienensis* showed a closer relationship with the family Varroidae, which made us to doubt whether *E*. *fukienensis* belongs to the family Laelapidae. Since only one species of *Echinolaelaps* was involved in this study and only a few species of the superfamily Dermanyssoidea were studied in this study, it is not enough to strongly support taxonomic status of *E*. *fukienensis*. However, taxonomic status of *E*. *fukienensis* or *V*. *destructor* should be problematic from results of genetic distance and phylogenetic tree. Therefore, it is hoped that obtain mitogenomes from more species of the superfamily Dermanyssoidea in the future. Such data would help to better understand phylogenetic relationships of the superfamily Dermanyssoidea.

## Conclusions

Structure and evolution of the *E*. *fukienensis* mitogenome have been analyzed for the first time, and provided insight into evolutionary relationships of different category level of the superfamily Dermanyssoidea. The *E*. *fukienensis* mitogenome was known the smallest in the superfamily Dermanyssoidea, with 37 genes typical of metazoan, including 13 protein-coding genes, 22 tRNA genes, 2 rRNA genes and 1 control region. The heavy strand encodes 22 genes and the light strand encodes 15 genes. Most PCGs have ATN as the start codon and TAN as the stop codon. However, unlike published structure of Gamasida, AT and GC skew values of *atp8* gene of *E*. *fukienensis* were both 0, it is rare in Gamasida. The average length of 22 tRNA genes of *E*. *fukienensis* is 64 bp, which is slightly smaller than the average length of 66 bp in arthropod tRNA genes, and there are base mismatches and missing the D-arms in tRNA secondary structures. Microsatellite like sequences of (AT) n and multiple palindromic sequences were present in control region of the *E*. *fukienensis* mitogenome. Phylogenetic tree supports the monophyly of the superfamily Dermanyssoidea. To obtain a more reliable phylogenetic tree, we need to collect and sequence more representative species of the superfamily Dermanyssoidea and further explore evolutionary mechanism of the superfamily Dermanyssoidea. These mitogenomes will provide novel molecular markers for studying the taxonomy and phylogeny of the superfamily Dermanyssoidea in the future.
