# Correction: The complete mitochondrial genome of *Echinolaelaps fukienensis* provide insights into phylogeny and rearrangement in the superfamily Dermanyssoidea

**DOI:** 10.1371/journal.pone.0305373

**Published:** 2024-06-06

**Authors:** Gangxian He, Wei Li, Bili Yuan, Wenge Dong

In Fig 2, the control regions should have been one (CR) not two (CR1 and CR2). Please see the correct Fig 2 here.

**Fig 2:**
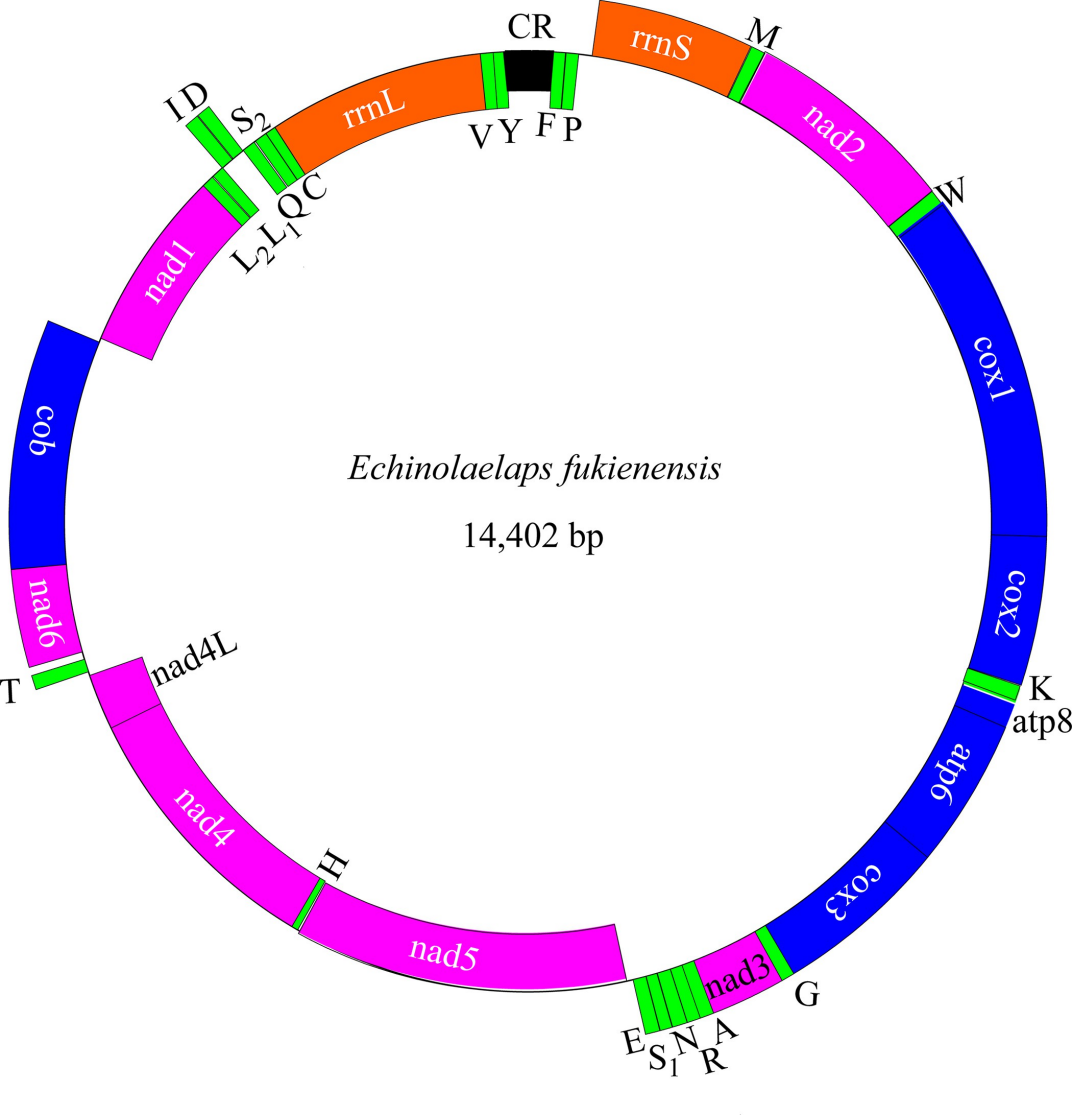
The complete mitogenome of *Echinolaelaps fukienensis*. Genes on the outside of the circle are coded on the major or J strand, whereas genes on the inside of the circle are on the complement (minor or N) strand.
